# Integrating machine learning and genome editing for crop improvement

**DOI:** 10.1007/s42994-023-00133-5

**Published:** 2024-02-29

**Authors:** Long Chen, Guanqing Liu, Tao Zhang

**Affiliations:** 1 Jiangsu Key Laboratory of Crop Genomics and Molecular Breeding/Zhongshan Biological Breeding Laboratory/Key Laboratory of Plant Functional Genomics of the Ministry of Education, Agricultural College of Yangzhou University, Yangzhou, 225009 China; 2 Jiangsu Co-Innovation Center for Modern Production Technology of Grain Crops/Jiangsu Key Laboratory of Crop Genetics and Physiology, Yangzhou University, Yangzhou, 225009 China

**Keywords:** Machine learning, Genome editing, Crop improvement, Molecular design breeding

## Abstract

Genome editing is a promising technique that has been broadly utilized for basic gene function studies and trait improvements. Simultaneously, the exponential growth of computational power and big data now promote the application of machine learning for biological research. In this regard, machine learning shows great potential in the refinement of genome editing systems and crop improvement. Here, we review the advances of machine learning to genome editing optimization, with emphasis placed on editing efficiency and specificity enhancement. Additionally, we demonstrate how machine learning bridges genome editing and crop breeding, by accurate key site detection and guide RNA design. Finally, we discuss the current challenges and prospects of these two techniques in crop improvement. By integrating advanced genome editing techniques with machine learning, progress in crop breeding will be further accelerated in the future.

## Introduction

Machine learning has brought about a revolution in various research domains, including agriculture. It is increasingly being utilized to enhance an understanding of crop growth and development, as well as to devise novel strategies for sustainable farming practices. Genome editing in crops is considered one of the most promising approaches to precision breeding, as it enables modification of plant DNA sequences with the goal of enhancing crop yields, stress tolerance, resistance to pests and diseases, nutritional quality, and more. However, genome editing in crop is a complex and challenging task that necessitates precise control over multiple parameters, such as the enzymes, targets, and editing results. Through the advancement of machine learning and its extensive implementation, researchers are able to ascertain the requisite parameters during genome editing procedures and predict editing outcomes, thereby facilitating the refinement of the editing system to enhance efficacy and attain desired outcomes.

Here, we offer a comprehensive overview of the recent progress in the application of machine learning to crop genome editing. This review mainly concentrates on three primary areas: optimizing genome editing systems with machine learning, exploring the synergy between genome editing and machine learning in crop breeding, and discussion of the prospects and challenges in crop improvement.

## Genome editing

### The evolution of genome editing

Genome editing is a technology for purposefully modifying the genome of organisms, including insertion, deletion, replacement and other mutations to DNA, resulting in altered biological traits. To edit specific loci or DNA segments in organisms, various genome editing technologies have been developed, such as Meganuclease, Zinc-Finger Nucleases (ZFNs), Transcription-Activator Like Effector Nucleases (TALENs) and clustered regularly interspaced short palindromic repeat (CRISPR)-associated protein (Cas) technology.

Meganucleases, which are natural endodeoxyribonucleases, possess an ability to recognize and cleave DNA sequences, typically spanning 20–30 base pairs, and allow site-directed genome modification, via re-engineering its DNA recognition site (Ashworth et al. 2006; Grizot et al. 2009; Jacquier and Dujon 1985). However, it is important to note that the process of reconfiguring the entire structure of a protein is time-consuming and challenging to implement, in practice, thus restricting their applications in genome editing.

Previous research clarified the role of zinc fingers in recognizing specific DNA sites and has successfully engineered zinc finger proteins (ZFPs) capable of identifying novel DNA sites (Desjarlais and Berg 1993). Consequently, this technology generates site-specific endonucleases, known as ZFNs, by fusing artificially designed ZFPs with the cleavage domain of the FokI endonuclease. This enables the identification of specific genome loci and facilitates targeted cleavage (Kim et al. 1996). Researchers are able to intentionally design ZFNs based on the target site, allowing them to recognize specific sequences and induce double-stranded DNA breaks (DSBs).

Transcription activator-like effectors (TALEs) are proteins secreted by *Xanthomonas* bacteria that can alter the transcription of genes in host plant cells. These effectors contain a central repetitive region, known as repeat variable di-residues (RVD), which consists of varying numbers of 34 amino acid repeat units. The RVD region is responsible for identifying specific DNA sequences and determining the biological specificity of each effector (Li et al. 2011). Following the principles of the ZFNs technique, TALENs are composed of FokI endonuclease domain and highly specific DNA-binding domains of TALEs, which are capable of inducing DSBs within a specific site (Christian et al. 2010).

Despite the successful achievement of genome editing, the intricate process of designing and constructing Meganucleases, TALENs and ZFNs has served to constrain their applications. Currently, the CRISPR technique stands out as the predominant genome editing method, due to its simplicity and high efficiency.

### CRISPR/Cas-based editing system

The CRISPR/Cas system is an adaptive defense mechanism present in archaea and bacteria, which utilizes small RNAs for precise detection and silencing of foreign nucleic acids (Barrangou et al. 2007). This system comprises a CRISPR array and a gene encoding a Cas protein with DNA cleavage activity. The CRISPR array consists of unique genome-targeting sequences (spacers) interspersed with identical repeats. Currently, various CRISPR/Cas systems have been discovered (Makarova et al. 2011, 2020). Among them, the most widely used and matured systems in the field of genome editing are the CRISPR/Cas9 and CRISPR/Cas12a. When confronted with viral and plasmid threats, the CRISPR/Cas9 system incorporate small fragments of foreign nucleic acids (protospacer) into the CRISPR locus in the host genome, leading to the formation of new spacers (Barrangou et al. 2007; Garneau et al. 2010). The CRISPR RNA (crRNA), originating from the CRISPR array and undergoing enzymatic cleavage, forms a complex with tracrRNA. This complex guides the Cas9 protein to recognize specific sequences in foreign nucleic acids to facilitates their cleavage. The target sequence recognized by the CRISPR/Cas system must contain the protospacer adjacent motif (PAM), which is essential for the editing system to identify binding sites and trigger the activation of the Cas protein for cleavage.

Building upon the CRISPR/Cas9 immune system, CRISPR/Cas9 technology employs a custom-designed single guide RNA (sgRNA) as a replacement for crRNA and tracrRNA, thus constructing a simplified editing system. Although the underlying principles are similar, CRISPR/Cas12a differs from CRISPR/Cas9 in sgRNA, protein and PAM, giving it unique features, including greater flexibility in target site selection (Zetsche et al. 2015).

Whether conventional CRISPR/Cas technology or ZFNs and TALENs, all are used to introduce DSBs, at specific sites, and activate cellular repair pathways, thereby enabling genome editing. Cellular repair pathways for DSBs primarily include non-homologous end joining (NHEJ) or homology-directed repair (HDR). HDR is a highly precise DNA repair mechanism that relies on the presence of a homologous template, allowing for precise gene insertion and correction (Feng Liang et al. 1998). Conversely, NHEJ joins the two ends of a DSB directly in the absence of a homologous template, frequently causing insertions or deletions and resulting in error-prone repair outcomes, thereby achieving gene knockout (Hartlerode and Scully 2009). Following the invention of CRISPR technology, five articles promptly highlighted its successful application in achieving gene knockout and modification in plants, demonstrating the powerful capabilities and enormous potential of CRISPR in plant biology (Feng et al. 2013; Li et al. 2013; Nekrasov et al. 2013; Shan et al. 2013; Xie and Yang 2013).

With the advancement of CRISPR system research and increasing demand for genome editing, several genome editing technologies derived from CRISPR have emerged. Among the most notable are base editing and prime editing. Base editing is accomplished by fusing a Cas9 nickase (nCas), or catalytically dead Cas (dCas), with an enzyme catalyzing a nucleobase deamination reaction, enabling the replacement of a target base without generating double-stranded breaks (Komor et al. 2016). Prime editor consists of a fusion protein, formed by dCas and reverse transcriptase (RT), along with a prime editing guide RNA (pegRNA). After the pegRNA guides the fusion protein to the specified site, it also serves as a template for reverse transcription, ultimately achieving the desired editing result at the target site (Anzalone et al. 2019). These emerging technologies have been successfully applied in various plant species, enabling precise editing and, thus, contributing to crop improvement (Li et al. 2017, 2018; Lin et al. 2020b).

### The significance of genome editing in crop breeding

Genome editing has revolutionized the study of gene function in crops by providing precise and selective manipulation of specific genes. This knowledge forms a solid foundation for crop breeding, allowing breeders to utilize genome editing for targeted modifications of genes associated with desired traits, through techniques such as targeted knockout, insertion, and replacement (Kim et al. 2019; Li et al. 2016; Wu et al. 2022a). By utilizing genome editing to modify *cis*-regulatory elements (CREs), such as promoters and enhancers, breeders are able to fine-tune gene expression levels, thereby enhancing traits (Zhang et al. 2018; Zhou et al. 2023). Harnessing genome editing to improve traits enables breeders to accomplish a myriad of goals in crops, including but not limited to increasing crop yield, improving nutritional content and quality, and enhancing resistance to various diseases and pests (Huang et al. 2020; Lu et al. 2018; Shi et al. 2017; Xu et al. 2021b).

Genome editing technology offers a promising approach to accelerating the breeding process beyond crop improvement. Unlike traditional breeding methods that rely on crossing or backcrossing, direct genome editing in elite varieties allows for the rapid development of superior varieties with targeted traits. This approach eliminates the need for time-consuming backcrossing and, thus, can significantly reduce the overall breeding time (Wulff and Dhugga 2018). Furthermore, the advantage of CRISPR in multiplex editing allows engineers to edit multiple gene loci simultaneously in a single generation (Yang et al. 2017).

## Machine learning

### Introduction to machine learning

**Fig. 1:**
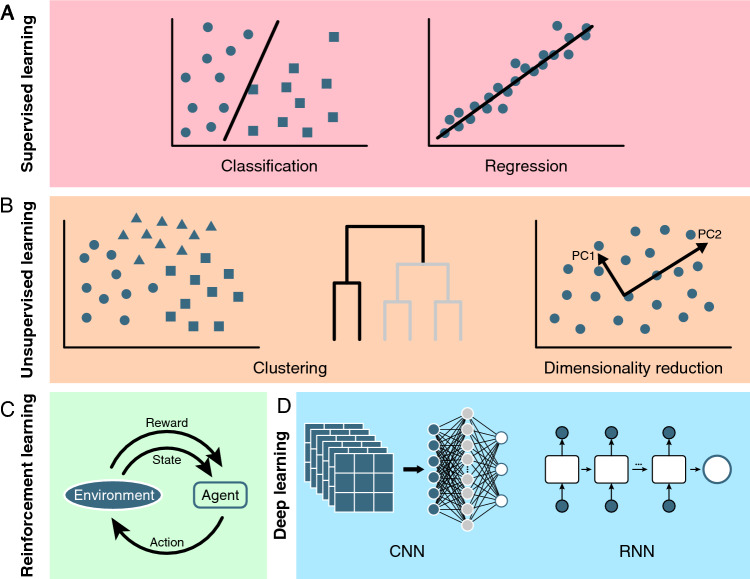
Classification of machine learning. **A** Supervised learning. Supervised learning utilizes labeled data to train models, allowing them to classify inputs into predefined categories (classification) or predict numerical values (regression). In a classification diagram, circles and squares represent samples with different labels, while lines represent the trained classification model. In a regression diagram, the circles represent labeled sample data, while the straight line represents the regression model’s fitted line. **B** Unsupervised learning. Unsupervised learning processes unlabeled data to group similar items (clustering) or simplify complex data for easier analysis (dimensionality reduction). Clustering algorithms analyze the similarity between samples, grouping similar samples into the same category to form clusters by groups (left) or by hierarchy (middle). In the diagram of dimensionality reduction, circles represent data points in high-dimensional space, and the two diagonal lines indicate the projection direction in the process of dimensionality reduction. **C** Reinforcement learning. The agent selects an action based on the observed current state, then receives rewards and a new state from the environment, adjusting its strategy to maximize future rewards in interactions. **D** Deep learning. CNN is primarily used for image recognition and processing. It detects features in images through convolutional and pooling layers, where filters in the convolutional layer capture local features, and the pooling layer reduces data dimensions. RNN, on the other hand, is a class of deep learning models suitable for sequential data like text and speech. Its key feature is the introduction of a recurrent structure, enabling it to memorize past information, making it highly effective in processing time-series data

Machine learning, a branch of artificial intelligence, enhances computer system performance by emulating human learning processes, through algorithms and data, allowing models to automatically learn patterns and rules from input data and make predictions or decisions when faced with unseen data. Machine learning can be categorized into several types: supervised learning, unsupervised learning, semi-supervised learning, and reinforcement learning (Fig. 1). Supervised learning involves training a model on labeled data, where the correct output is known in advance. The model learns the relationship between inputs and outputs, enabling it to predict outputs for new inputs. Supervised learning algorithms can be further divided into classification and regression (Table 1). Classification algorithms are used to predict or categorize distinct values, such as diagnosis of crop diseases (Jiang et al. 2020). Regression algorithms are primarily used for analyzing and predicting continuous numerical data, such as crop yield prediction (Pantazi et al. 2016).

**Table 1:** Common machine learning algorithms

Type	Algorithms
Supervised learning	Classification: Logistic Regression, K nearest neighbors, Naive Bayesian
	Regression: Linear Regression, Ridge Regression
	Both regression and classification: Decision tree, Support Vector Machine (SVM), Random Forest
Unsupervised learning	Clustering: K-means Clustering, Hierarchical Clustering
	Dimensionality reduction: Principal Component Analysis (PCA), Linear Discriminant Analysis (LDA)
Semi-supervised learning	Transductive SVM, Generative Models, Self-Training
Reinforcement learning	Q-Learning, State-Action-Reward-State-Action, Deep Q-networks

Unsupervised learning trains a model on unlabeled data, where the correct output is unknown beforehand. It focuses on discovering inherent structures and patterns within data. Unsupervised learning mainly includes clustering and dimensionality reduction (Table 1). Clustering is the process of grouping objects with similar features into the same category, whereas dimensionality reduction involves reducing the feature dimensions of a dataset while preserving its essential characteristics. Semi-supervised learning is a combination of supervised and unsupervised machine learning methods, aiming to enhance model performance by utilizing limited labeled data alongside a large amount of unlabeled data (Table 1). Reinforcement learning involves training a model through trial-and-error, with rewards or penalties given for certain actions (Table 1).

Deep learning, a recent focus in the field of machine learning, utilizes artificial neural networks (ANNs) that replicate the structure and functions of the human brain. By using multi-layered neural networks, such as convolutional neural networks (CNN), recurrent neural networks (RNN), long short-term memory networks (LSTM) and transformer, deep learning can automatically extract abstract features from the data through layer-by-layer learning. This capability enables deep learning to excel at handling large-scale and high-dimensional data.

In the machine learning, both features and labels are pivotal as they furnish the model with the necessary information to facilitate predictions or decisions. Features refer to the characteristics or properties of an individual that are used to make predictions or decisions. Labels, on the other hand, are values or categories assigned to individuals or items, representing their identity or classification. In the field of crop science, the features of an individual crop may encompass various aspects, such as size, shape, color, texture, and other relevant traits. Labels in this context might indicate the type of plant, such as “corn”, “wheat”, or “soybean”. A practical application of machine learning involves constructing models capable of accurately predicting the label of new plants based on their features. For example, Saleem et al. extracted shape and texture features from diverse plant leaf images. Using plant varieties as labels and leaf characteristics as features, they developed a machine learning-based model, which has the capacity to predict unknown plant varieties dependent on leaf images (Saleem et al. 2019).

### Applications of machine learning to plants

Developing a model for plant species classification is just one among the diverse applications of machine learning in the field of plant sciences. In addition to this application, machine learning has extensive use in various areas, including plant genomics, proteomics, metabolomics, and phenomics. Machine learning can assist in unraveling the complexity and diversity of plant genomes. Due to advances in sequencing technology and bioinformatics algorithms, genome assembly has become relatively simple. However, identifying functional regions within the genome remains challenging. Machine learning offers a solution by enabling the identification of structural regions in the genome, particularly focusing on protein-coding genes and *cis*-regulatory elements (CREs). Several software tools based on machine learning algorithms, such as Hidden Markov Models (HMM) and Support Vector Machine (SVM), have been developed for ab initio gene prediction using information from genomic sequences (Korf 2004; Schweikert et al. 2009; Stanke et al. 2006). The utilization of AUGUSTUS, for instance, enables accurate identification and annotation of genes in unknown genomes with the aid of a trained annotation file, providing vital insights for genome research. Convolutional neural networks (CNNs) are widely employed in predicting *cis*-regulatory elements by learning patterns and features in non-coding regions (Umarov and Solovyev 2017). Moreover, machine learning is utilized for constructing gene regulatory networks and predicting gene–gene interactions, which helps researchers to discover intricate relationships within the genome (Haque et al. 2019).

The function of machine learning in plant proteomics lies in promoting a deep understanding of protein structure, function, and interactions. The emergence of AlphaFold2 undoubtedly represents a highly successful application of machine learning in the field of proteomics research (Jumper et al. 2021). AlphaFold2 utilizes a machine learning approach to predict the structure of proteins, with atomic-level accuracy, based solely on its amino acid sequence. This breakthrough significantly reduces the time and effort required for studying protein structure and function. Ziyun Ding and Daisuke Kihara developed a computational method that utilizes known protein–protein interactions (PPIs) data from *Arabidopsis thaliana* for training and testing (Ding and Kihara 2019). This method takes into account various features, including protein sequences and gene co-expression, and can be applied to other crops to discover new PPIs on a genome-wide scale.

Machine learning in metabolomics contributes to elucidating the mechanisms of plant growth, adaptation, and responses to the environment. Compared to traditional biochemical and genetic methods, using machine learning to predict metabolic pathways in plants not only accelerates the research process but also helps explore new pathway memberships and related genes (Moore et al. 2020).

The application of machine learning to extract useful information from images and videos is becoming a key technique for identifying phenotypic changes in plants. High-throughput phenotyping aided by machine learning for image analysis will play an essential role for crop yield improvement. By collecting and analyzing plant trait data, such as seeds, leaves, and roots, these techniques enable the classification of mutants within the same species as well as different species (Lo Bianco et al. 2017; Pound et al. 2017; Rzanny et al. 2017). Using machine learning techniques, especially in the field of computer vision, such as image processing and pattern recognition, rich phenotypic features, such as color, shape, and texture, can be extracted from plant images. These features aid in detecting and classifying plant diseases, enabling early and accurate identification of disease types, and guiding farmers to take appropriate measures for prevention and control (Khan et al. 2022; Shrivastava and Pradhan 2021). Besides, machine learning algorithms are also able to predict crop yields more accurately by analyzing vast amounts of agricultural data, including meteorological data, soil information, and field management (van Klompenburg et al. 2020).

## Optimizing CRISPR/Cas editing systems with machine learning

**Fig. 2:**
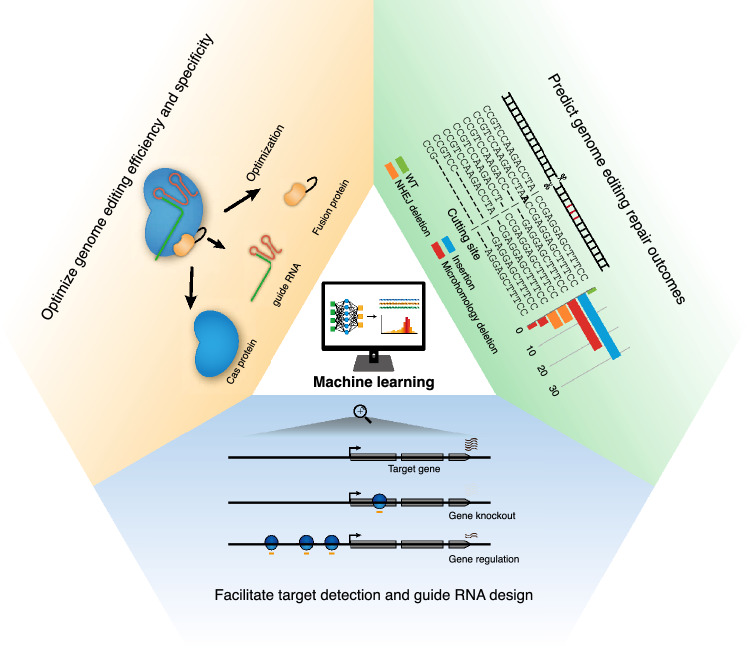
The application of machine learning in genome editing. Machine learning uses vast datasets and computation to optimize genome editing systems and guide their usage from three main aspects: optimize genome editing efficiency and specificity by designing robust guide RNAs, digging out new Cas orthologs and variants, substitutable fusion proteins; Predict genome editing repair outcomes by assessing diverse repair mechanisms from plenty of repair results using machine learning; Facilitate target detection and guide RNA design by integrating muti-omics data into machine learning to discover critical genes and mine key sites

With the rising application of genome editing technology in breeding, there is a growing need for more efficient and precise CRISPR editing. However, the challenges, such as the inconsistency in CRISPR editing efficiency and outcomes, along with persistent off-target effects, remain as significant hurdles. To address these issues, designing and optimizing CRISPR/Cas editing systems has become a crucial strategy. By leveraging machine learning algorithms to analyze extensive experimental data, researchers identify patterns and regularities within the dataset, which in turn guides them in optimizing editing systems, thereby paving the way for more effective and precise genome editing (Table 2, Fig. 2).

**Table 2:** Applications of machine learning in optimizing genome editing systems and crop breeding

Areas	Directions	Applications of machine learning
Optimizing genome editing systems	Predicting the impact of different guide RNAs on editing efficiency	Rule Set 1, Rule Set 2, Rule Set 3, FORECasT-BE, DeepPE
	Predicting the impact of different Cas variants on editing efficiency	DeepSpCas9variants, DeepSmallCas9
	Designing high-specificity guide RNA	Elevation, DeepCRISPR, CnnCrispr
	Optimizing editing protein	AlphaFold, AlphaFold2, RoseTTAFold
	Predicting genome editing outcomes	FORECasT, SPROUT, inDelphi, CROTON
Crop breeding	Gene discovery and prioritization	GRAiN, QTG-Finder, QTG-Finder2
	Mining cis-regulatory elements and key sites	iCREPCP

### Optimizing editing efficiency

#### Predicting the impact of different guide RNAs on editing efficiency

The efficiency of the CRISPR/Cas system in editing varies significantly depending on the target site, and numerous studies have been conducted to identify factors that influence editing efficiency. Previous studies have shown that the characteristics of the guide RNA and target site, such as nucleotide composition, GC content, RNA secondary structure, and epigenetic states of the target, are associated with editing efficiency (Doench et al. 2014, 2016; Szczelkun et al. 2014). Since these factors function in complex ways, and their underlying rules are not entirely elucidated by experimental observations, designing optimal sgRNAs for specific genome editing remains a formidable challenge. To overcome this challenge, machine learning approaches have emerged as powerful tools to construct predictive models, based on these features, thereby enhancing the ability to forecast efficiency and select high-efficiency sgRNAs.

Doench and his colleagues systematically investigated the optimization of sgRNA, design by constructing multiple sgRNA libraries, in human and mouse, and leveraging the large-scale experiment data to build machine learning predictive models (Table 3). Initially, they took the sequence features into consideration and developed an on-target model named Rule Set 1 (Doench et al. 2014). To further improve the predictive model, they then doubled the size of the sgRNA activity data set and introduced thermodynamic properties to generate a Rule Set 2 model of CRISPR-Cas9 on-target efficiency, which showed an elevated performance compared to the first version (Doench et al. 2016). With the advent of new datasets, features and model architectures, Rule Set 2 has the possibility of further improvement. Consequently, Rule Set 3 was developed with larger datasets and tracrRNA identity feature, and trained with a gradient boosting model (DeWeirdt et al. 2022). Rule Set 3 is superior to previous models in sgRNA activity predictions, suggesting the expanding experimental datasets and features will strengthen the ability of models to predict guide RNA efficiency.

**Table 3:** Comparison of Rule Set 1–3 models

Model	Datasets	Algorithms	Features
Rule Set 1	1841 sgRNAs for 9 genes	Logistic regression	Single nucleotide, dinucleotide, GC content
Rule Set 2	4390 sgRNAs for 17 genes	Gradient-boosted regression trees	All features in Rule Set 1, nucleotide count, PAM sequence, thermodynamics
Rule Set 3	46,526 unique context sequences from 7 datasets	Gradient boosting framework from LightGBM	All features in Rule Set 2, tracrRNA identity

Due to the different functionalities, the editing efficiency varies a lot among different genome editing systems. Therefore, several specialized tools have been developed for base editor (BE) and prime editor (PE), such as FORECasT-BE (Pallaseni et al. 2022) and DeepPE (Kim et al. 2021). These models are built with intrinsic features from specific editing systems and efficiency data from high-throughput assessments. Although a series of models and tools have been developed for predicting editing efficiency and designing guide RNAs, their individual performances show great differences on the same experimental dataset, which are likely attributed to differences in feature selection, dataset usage, and algorithmic approaches (Haeussler et al. 2016).

#### Predicting the impact of different Cas variants on editing efficiency

The efficiency of genome editing is not solely determined by sgRNAs, but also influenced by the inherent nuclease activity. The Cas9 protein, which is most commonly used, namely from *Streptococcus pyogenes* (SpCas9), may sometimes be limited by off-target effects and the absence of a specific PAM sequence at the target site. To realize specific editing requirements, researchers have discovered many Cas orthologs and developed a series of Cas variants with distinct characteristics compared to traditional Cas proteins. As an example, Kim et al (2020) selected 13 Cas variants with high specificity and/or broad PAM compatibility and evaluated their cleavage efficiency across 26,891 target sequences. They used the insertion and deletion (indel) frequency data, obtained from matched target sequences with PAM sequences, as training and testing datasets. Their investigation unveiled those variations in target sequence preferences among different Cas variants that resulted in divergent editing efficiencies within the same target region. To accurately predict the activity of different Cas variants, at different sites, a CNN deep learning model, named DeepSpCas9variants, was developed. Similarly, Kim and coworkers also systematically compared the efficiencies of 17 small Cas9 variants at numbers of target sites and developed a corresponding deep learning-based predictive model, called DeepSmallCas9 (Seo et al. 2023).

The integration of machine learning enables researchers to gain deeper insights into the characteristics of diverse Cas variants and accurately predict their efficacy on target sequences. This progress equips researchers with an ability to select a Cas that is engineered for specific applications, thereby enhancing the effectiveness of genome editing.

### Optimizing editing specificity

#### Designing high-specificity guide RNA

Similar to a PCR primer, guide RNA may also bind to non-target sites with minor mismatches and result in unexpected cleavage, known as off-target effects, although the editing sites of CRISPR/Cas system are strictly restricted by the guide RNA and PAM (Hsu et al. 2013; Zhang et al. 2015). Previous studies have demonstrated that prediction of CRISPR cleaving specificity and designing highly specific sgRNA can effectively reduce the occurrence of off-target effects (Hsu et al. 2013).

Several software or platforms that are currently being developed for predicting off-targets generally rely on three approaches, including alignment-based methods [Cas-OFFinder (Bae et al. 2014), CasOT (Xiao et al. 2014)], hypothesis-driven methods [CRISPOR (Concordet and Haeussler 2018), CHOPCHOP (Labun et al. 2016)] and learning-based methods [Elevation (Listgarten et al. 2018), DeepCRISPR (Chuai et al. 2018), CnnCrispr (Liu et al. 2020)]. Compared to other approaches, learning-based methods may better predict guide RNA specificity by building complex models using combinations of multiple features.

The learning-based methods for predicting guide RNA specificity typically involve three steps. First, all potential off-target sites of guide RNA are scanned across the entire genome. Second, machine learning models that score potential off-target sites are built, based on multiple features such as sequence features and chromatin accessibility, since these potential off-target sites may not actually be edited. Third, a single numeric score, which integrates individual target scores, is generated for evaluating the specificity of guide RNA by constructing a model or specific algorithms. Listgarten et al. (2018) developed Elevation, a two-layer regression model that utilizes the seed and extension method to identify near-matching CRISPR-Cas9 target sites as potential off-target sites, with scores integrated through Elevation-aggregate, a gradient boosted regression tree model.

Based on extensive experiments various methods have been developed to detect off-target sites, such as GUIDE-seq, Discover-seq, and CIRCLE-seq (Tsai et al. 2015, 2017; Wienert et al. 2019). By utilizing these methods, researchers are afforded a large dataset of off-targets for training the model. DeepCRISPR, a hybrid neural network model, utilizes genome-wide unlabeled sgRNA sequences and corresponding epigenetic information to encode a pre-trained network. Then off-target data from experiments were then gathered to fine tune the pre-trained network and a CNN classifier was concatenated to predict the off-target sites (Chuai et al. 2018). In contrast to conventional machine learning approaches, deep learning algorithms are capable of managing larger volumes of data and automatically extracting intricate features from the input data, ultimately leading to more precise predictive outcomes.

#### Optimizing the editing protein

Apart from designing highly specific guide RNAs, another approach to reduce off-target effects includes the modification of editing proteins, or the investigation of new proteins. The use of evolutionary and bioinformatics methods facilitates the identification of novel editing proteins, such as LrCas9 (Zhong et al. 2023), Cas13X, and Cas13Y (Xu et al. 2021a). Furthermore, by applying protein engineering methods, such as directed evolution and rational design, existing editing proteins can be effectively optimized, such as VQR-Cas9 (Kleinstiver et al. 2015b), SpRY (Walton et al. 2020), xCas9 (Hu et al. 2018), SpCas9-NG (Nishimasu et al. 2018), eSpCas9 (Slaymaker et al. 2016), LZ3 Cas9 (Schmid-Burgk et al. 2020)  SaKKH-Cas9 (Kleinstiver et al. 2015a). Compared to the widely used SpCas9, these novel editing proteins and engineered editing proteins, respectively, demonstrate advantages in terms of size, PAM, editing efficiency and specificity. In recent years, several systems derived from such proteins have been successfully applied in plant genome editing, showcasing their potential in enhancing precision and efficacy (Hua et al. 2019; Li et al. 2021a; Ren et al. 2019; Wang et al. 2019).

The traditional methods for discovering and optimizing proteins may face challenges such as extensive time and cost requirements. Furthermore, the construction of high-fidelity Cas9 variants necessitates existing structural and/or functional information to select and mutate amino acid residues that could influence the specificity of Cas cleavage (Bravo et al. 2022). However, the recent advancements in machine learning technology, particularly the emergence of protein structure prediction tools like AlphaFold (Senior et al. 2020), AlphaFold2 (Jumper et al. 2021), and RoseTTAFold (Baek et al. 2021), have significantly enhanced the accuracy of protein structure and function predictions. This has made the refinement of Cas proteins more efficient and has provided valuable insights into protein-substrate interactions and identified potential mutational hotspots. The AlphaFold2 was employed to predict and compare the structures of wild-type Cas12a and three active variants, revealing a transition from a closed to an open conformation. This finding was advantageous for elucidating the impact of mutations on protein structure and function, establishing the groundwork for designing and engineering highly specific Cas variants (Ma et al. 2022).

In base editing, apart from off-target effects associated with guide RNA and Cas protein, deaminase enzymes may also cause off-target editing, independent of the sgRNA (Sretenovic et al. 2023; Wu et al. 2022b). Cytosine base editors (CBEs) were also shown to induce genome-wide off-target mutations in rice (Jin et al. 2019). The deaminase like rAPOBEC1 used in CBEs binds to single-stranded DNA independent of Cas9 and sgRNA, leading to off-target single nucleotide variants (SNVs). Machine learning approaches are suitable for fast discovery of deaminases with lower off-target activities. Huang et al. (2023) selected proteins with a deaminase domain and utilized AlphaFold2 to predict protein structures from their sequences. Subsequently, they clustered these proteins into different clades, with specific functions, based on structural similarity. After experimental validation, the SCP1.201 clade was shown to comprise various new proteins capable of performing single-stranded DNA deamination, enabling their application in base editing. Among the newly discovered deaminases, several exhibited lower off-target activities than rAPOBEC1. Such studies reveal the power of machine learning to rapidly identify the functions and characteristics of new proteins, thereby facilitate the discovery of deaminases better suited for base editing. This approach opens new possibilities for the development of more efficient and precise genome editing tools, contributing to the advancement and application of genome editing technology to crop breeding programs.

### Predicting genome editing outcomes

In conventional CRISPR/Cas9 genome editing, the double-stranded breaks generated by CRISPR are mainly repaired by the template-free non-homologous end joining (NHEJ) and microhomology-mediated end joining (MMEJ). Despite NHEJ and MMEJ introducing mutations during the repair process, these repair outcomes are nonrandom and foreseen (Chakrabarti et al. 2019; Molla and Yang 2020). Previous studies have demonstrated that repair outcomes are influenced by various DNA sequence features, such as GC content, microhomology length, and position. Consequently, a series of machine learning models have been developed for predicting genome editing outcomes (Allen et al. 2019; Leenay et al. 2019; Shen et al. 2018) (Fig. 2). Molla and Yang (2020) summarized three different machine learning models [(FORECasT (Allen et al. 2019), SPROUT (Leenay et al. 2019) and inDelphi (Shen et al. 2018)] that predict Cas9 repair outcomes in human cells. These models demonstrated remarkable accuracy in forecasting both insertions and deletions.

Although trained on experimental data from mammalian cells, these models may be adopted for predicting repair outcomes in plants, as mutations depend heavily on the DNA sequences surrounding DSBs. Applying these models for predicting editing outcomes in plants will help design experiments for precise genome editing. Limited knowledge of the repair mechanisms during CRISPR/Cas9 editing restricts the selection of appropriate features for building models. Therefore, a deep learning model called CROTON was developed (Li et al. 2021b), which utilizes deep multi-task CNNs and neural architecture search (NAS). CROTON automates both feature and model engineering to predict editing outcomes directly from raw sequences with higher accuracy. Machine learning helps researchers forecast the possible outcomes, facilitate the experiments design and speed up the subsequent validation.

## Machine learning bridges crop genome editing and breeding

Discovery of agronomically utilizable genes and prioritization of important gene resources are vital for crop breeding. Utilizing genome editing techniques to manipulate these genes can effectively improve a crop in many areas, such as yield, pathogen resistance, or nutrition. Computational approaches, especially machine learning, play an important role in mining the breeding-related genes and detecting the key elements and factors that regulate the expression of these genes (Table 2).

### Gene discovery and prioritization

Due to the complex regulatory relationships among genes and their associations to traits, identifying trait-related genes can be a laborious task. Integrating and analyzing multiple omics data with machine learning techniques accelerates the process of gene discovery and prioritization (Zhang et al. 2016; Zhao et al. 2020). This approach leverages gene regulatory networks (GRNs) and genome-wide association studies (GWAS), which enable the screening of candidate trait-related genes and their regulatory relationship, to identify potential targets for genome editing (Bao et al. 2023).

A Gene Regulation and Association Network (GRAiN) of rice and a supervised machine learning framework was developed identify and rank transcription factors (TFs) and genes related to drought resistance (Gupta et al. 2021). This approach successfully predicted a transcription factor, OsbHLH148, and its associated genes, which play a crucial role in drought resistance. Compared to GRN, identifying trait-related genes through GWAS is more intuitive because it directly associates specific genotypes with traits. QTG-Finder prioritizes causal genes in quantitative trait loci (QTLs) by learning features from known causal genes in *Arabidopsis* and rice, including DNA polymorphisms and functional annotations (Lin et al. 2019). Building upon this, QTG-Finder2 integrates orthologous information from various plant species, thereby expanding the applicability range (Lin et al. 2020a). With QTG-Finder2 and public transcriptome data, researchers set, as a priority, a plant height QTL, and identified 13 candidate genes, including the green revolution gene, *SD1*; editing of these candidates reduced plant height, while moderately increasing yield, rendering it a valuable candidate for breeding.

### Mining cis-regulatory elements and key sites

Gene expression is regulated by the interaction between trans-acting factors and cis-regulatory DNA elements (CREs). Previous studies have substantiated the efficacy of manipulating CREs in enhancing crop traits. Specifically, genome editing of CREs within the *ARGOS8* promoter region in maize was shown to confer increased yield under drought stress conditions (Shi et al. 2017). Similarly, genome editing of promoters in tomato has led to the generation of a diverse array of cis-regulatory alleles that confer improved traits, thereby offering significant advantages for breeding purposes (Rodriguez-Leal et al. 2017). This editing of promoter regions generated diverse cis-regulatory alleles, providing beneficial quantitative variation for breeding.

Machine learning serves as a versatile approach that integrates multi-omics data, to efficiently identify CREs and key nucleotide residues for genome editing. Akagi and colleagues construct an interpretable convolutional neural network (CNN) model, by using cistrome datasets, to predict genome-wide expression patterns in tomato and then experimentally validated the impacts of the predicted key sites on fruit ripening initiation by mutating the nucleotide residues (Akagi et al. 2022). Additionally, iCREPCP, a deep learning-based platform, identifies critical CREs in the plant core promoter sequences with base-level resolution, thereby providing important candidate targets for genome editing (Deng et al. 2023). Zhou et al. (2023) combined regression-based methodology with empirical knowledge to develop a weighted average prediction algorithm, which is able to estimate the potential impact of editing different regions of the promoter on gene expression. By combining this model with CRISPR-Cas12a promoter editing, they successfully developed a series of useful quantitative trait variation continuum in crops, including mutants carrying a quantitative green revolution trait. Through implementing machine learning, researchers are able to predict crucial nucleotide positions highly relevant to gene expression. By editing these key sites, using genome editing technologies, breeders are afforded the opportunity to engineer new alleles for optimal gene expression, ultimately achieving precise control of gene expression to obtain ideal phenotypic outcomes.

Once the targets for genome editing have been determined, breeders attempt to implement precise modifications, at designated loci, to attain the desired traits and facilitate precision breeding. CRISPR-derived technologies, like base editing and prime editing, have been successfully applied in targeted improvement of various traits across a broad spectrum of crops (Butt et al. 2020; Li et al. 2018; Mao et al. 2019). As mentioned earlier, machine learning has been utilized to optimize genome editing systems and predict editing outcomes, ensuring that the modifications to the genome align with expectations. The integration of machine learning into genome editing processes has strongly propelled the development of precision breeding capabilities.

## Future prospects

### Epigenetics, machine learning and genome editing

Epigenetics refers to heritable and stable changes in gene expression that occur without alterations to the DNA sequence, involving DNA methylation, histone modifications, and non-coding RNA regulation (Berger et al. 2009). Epigenetic mechanisms, which regulate gene expression and influence traits by chemically modifying DNA bases or altering the chromosomal superstructure, will be targeted for modification using CRISPR-based epigenome editing. This is achieved by the fusion of dCas9 with catalytic domains from a chromatin-modifying enzyme to modify epigenetic marks at specific genomic loci, resulting in target gene up-regulation or down-regulation (Chavez et al. 2015; Gilbert et al. 2013; Konermann et al. 2015; Nunez et al. 2021; Zalatan et al. 2015).

As some of the most important crop agronomic traits are known to be controlled in an epigenetic manner, epigenome editing is expected to become an effective breeding method. Epigenome editing technology was successfully applied to *Arabidopsis* to obtain promising breeding traits, such as suitable flowering time and drought stress resilience (Paixao et al. 2019; Papikian et al. 2019). In addition to discovering the epigenetic mechanisms regulating traits of interest, machine learning is utilized to optimize the epigenetic editing system by designing highly efficient guide RNA (Huang et al. 2022; Yang et al. 2023). Although current successful cases are mainly focused on model plants, epigenome editing, assisted by machine learning, will become a widely applicable and effective crop breeding method in the future.

### Single-cell technology, machine learning and genome editing

Single-cell technology refers to a research methodology that entails the experimental isolation of individual cells, followed by sequencing and multi-omics analysis, conducted at the cellular level. Single-cell CRISPR screening techniques, such as Perturb-Seq, CRISP-seq, or CROP-seq, combine pooled CRISPR screening with single-cell RNA-seq, enabling the investigation of functional CRISPR screening at a single-cell level (Datlinger et al. 2017; Dixit et al. 2016; Jaitin et al. 2016). The technique has been successfully applied in mammalian cells to analyze gene function, study cellular heterogeneity, and map gene regulatory networks (Fang et al. 2019; Rubin et al. 2019).

The adaptation of these techniques for plants has remained a challenge (Gaillochet et al. 2021). The heterogeneity in plant cell size and the requirement to digest cell walls greatly affect the efficiency of single-cell isolation, resulting in the number of harvested plant cells being insufficient for single-cell CRISPR screening (Shulse et al. 2019). To resolve this issue, the use of a machine learning framework might allow the number of cells analyzed to be downscaled to identify core regulatory signatures resulting from genetic perturbations (Dixit et al. 2016). As both CRISPR screens and single-cell sequencing have been established in plants (Lu et al. 2017; Tao et al. 2022), it seems reasonable that, in due course, single-cell CRISPR screens will be realized in plants. Furthermore, with the constant improvement of experimental pipelines and computational frameworks, genome editing, screening, and functional studies at single-cell level in plants will soon be achievable.

### Crop improvements by molecular design

Leveraging phenotypic data and multi-omics information, machine learning-based GWAS enhances our comprehension of the correlation between genomic variations and specific traits. This approach deepens our insights into the genetic underpinnings of complex traits, laying a robust theoretical foundation for advancements in molecular breeding (Yoosefzadeh-Najafabadi et al. 2022). Another potential path to provide biological knowledge and theoretical foundation for breeding in the future is intelligent breeding, based on state-of-the-art AI-powered language models. Here, we refer to the use of natural language processing (NLP) and machine learning algorithms to analyze extensive literature in botany, genetics, and biology. The goal is to comprehend the knowledge in plant genomics, predict interactions between genes, identify relationships between plant traits and genotypes, and offer breeding recommendations. Based on the acquired biological knowledge, breeders will efficiently and accurately achieve molecular-designed breeding through genome editing. With a continuous advancement of machine learning and genome editing technologies, our understanding of the plant genome will deepen, and the ability to modify the genome will become increasingly more powerful. This will enable breeders to design and cultivate ideal varieties with unprecedented flexibility and precision.

### Crop breeding by de novo domestication

As an innovative breeding approach, de novo domestication of wild relatives involves genetic editing to modify wild species, at the gene level, preserving their adaptation to complex environments and other beneficial traits, while ensuring their major economic traits match or surpass those of existing cultivated varieties (Zsogon et al. 2017). A recent study reported the rapid improvement of agronomically important traits in a wild allotetraploid rice, by editing homologs of genes controlling these traits in diploid rice, accomplishing de novo domestication (Yu et al. 2021). To expedite the process of de novo domestication, machine learning will be adopted to accelerate the identification of genes related to domestication traits. By comparing the genetic variations between wild types and their corresponding cultivated varieties, machine learning algorithms pinpoint key domestication genes. These identified genes serve as vital targets for genome editing, enabling the effective introduction of domestication traits. Furthermore, since these genes often have pleiotropic effects, and the outcomes of different combinations of edited alleles are difficult to predict, machine learning will assist in identifying the impacts of various gene combinations through analyzing vast genomic datasets. This helps scientists in making better-informed editing decisions to achieve the desired optimal traits. As machine learning continues to refine our understanding of genetic interactions, it opens new avenues for enhancing the efficiency and precision of de novo domestication, revolutionizing the future of crop breeding.

### Crop breeding through synthetic biology

Synthetic biology involves the design, engineering, or de novo synthesis of biological systems with specific functions, based on life science knowledge through engineering methods, targeting a diverse range of objectives including new genes and even entire new species. Genome editing is an effective method in synthetic biology that enables precise control of living organisms by rapidly and accurately altering their genes. As an example, prime editing was used to synthesize a novel allele, *Xa23SW14*, which imparts resistance to bacterial blight disease in rice (Gupta et al. 2023). In the future, genome editing technology will be harnessed to engineer, or synthesize, novel genes with tailored functionalities that are customized to meet individualized requirements, thereby transcending the constraints of conventional breeding. There are some cases in which genome editing induces two or more DNA double-stranded breaks, simultaneously, and the NHEJ and MMEJ mechanism may mediate the occurrence of chromosomal rearrangements (Rönspies et al. 2021b; Schmidt et al. 2020). Such chromosomal rearrangements may completely reshape the chromosomes of edited plants (possibly reducing the number of chromosomes), leading to reproductive isolation between the engineered line and the wild-type parent. In such a case, the engineered line may be considered as a new plant species (Rönspies et al. 2021a).

The uncertainty of editing results and side effects in the complex biological systems are challenges associated with applying synthetic biology to plants. Through the utilization of machine learning algorithms, it is possible to predict editing outcomes from data without a comprehensive comprehension of the fundamental principles, thereby facilitating the advancement of genome editing-based synthetic biology in crop breeding. Although synthetic biology operations can be arduous, the benefits it affords are incomparable to other breeding methods.

## Conclusion

Crop genome editing is a rapidly evolving field with promises to improving crop yield, enhancing nutritional content and resistance in crops. Machine learning is emerging as a powerful approach for automating and optimizing crop genome editing processes. Using machine learning not only optimizes editing systems, to achieve higher efficiency and specificity, but also assists breeders in identifying key functional region and sites in genome to realize precision breeding objectives.

By combining the best practices from both fields, the full potential of crop genome editing will be unleashed for improving traits of crops. In the future, integrating genome editing, machine learning, the latest advancements in biological technologies, and research findings will expand the functionalities and applications of genome editing and provide breeders with a powerful toolkit for crop improvement. Moreover, when machine learning and genome editing are applied in innovative breeding methods, such as molecular design breeding, de novo domestication and synthetic biology, it will surpass the capabilities of traditional breeding as well as usher in a revolution in modern agriculture.

## Data Availability

Data sharing not applicable to this article as no datasets were generated or analyzed in the study.
